# Metabolic heterogeneity in adrenocortical carcinoma impacts patient outcomes

**DOI:** 10.1172/jci.insight.167007

**Published:** 2023-08-22

**Authors:** Qian Wang, Na Sun, Raphael Meixner, Ronan Le Gleut, Thomas Kunzke, Annette Feuchtinger, Jun Wang, Jian Shen, Stefan Kircher, Ulrich Dischinger, Isabel Weigand, Felix Beuschlein, Martin Fassnacht, Matthias Kroiss, Axel Walch

**Affiliations:** 1 Research Unit Analytical Pathology and; 2 Core Facility Statistical Consulting, Helmholtz Zentrum München Deutsches Forschungszentrum für Gesundheit und Umwelt, Neuherberg, Germany.; 3 Institute of Pathology, and; 4 Division of Endocrinology and Diabetes, Department of Internal Medicine, University Hospital of Wuerzburg, Wuerzburg, Germany.; 5 Department of Internal Medicine IV, LMU Hospital, Ludwig-Maximilians-Universität München (LMU), Munich, Germany.; 6 Department of Endocrinology, Diabetology and Clinical Nutrition, University Hospital Zurich (USZ) and University of Zurich (UZH), Zurich, Switzerland.; 7 Comprehensive Cancer Center Mainfranken, University Hospital of Wuerzburg, Wuerzburg, Germany.

**Keywords:** Metabolism, Oncology, Bioinformatics, Cancer

## Abstract

Spatially resolved metabolomics enables the investigation of tumoral metabolites in situ. Inter- and intratumor heterogeneity are key factors associated with patient outcomes. Adrenocortical carcinoma (ACC) is an exceedingly rare tumor associated with poor survival. Its clinical prognosis is highly variable, but the contributions of tumor metabolic heterogeneity have not been investigated thus far to our knowledge. An in-depth understanding of tumor heterogeneity requires molecular feature-based identification of tumor subpopulations associated with tumor aggressiveness. Here, using spatial metabolomics by high–mass resolution MALDI Fourier transform ion cyclotron resonance mass spectrometry imaging, we assessed metabolic heterogeneity by de novo discovery of metabolic subpopulations and Simpson’s diversity index. After identification of tumor subpopulations in 72 patients with ACC, we additionally performed a comparison with 25 tissue sections of normal adrenal cortex to identify their common and unique metabolic subpopulations. We observed variability of ACC tumor heterogeneity and correlation of high metabolic heterogeneity with worse clinical outcome. Moreover, we identified tumor subpopulations that served as independent prognostic factors and, furthermore, discovered 4 associated anticancer drug action pathways. Our research may facilitate comprehensive understanding of the biological implications of tumor subpopulations in ACC and showed that metabolic heterogeneity might impact chemotherapy.

## Introduction

Adrenocortical carcinoma (ACC) is a rare and malignant tumor with up to 10% of early-stage tumors being incidentally diagnosed (1, 2). It is frequently referred to as an orphan disease. Currently, the clinical outcomes of patients with ACC are variable and difficult to predict. Some patients exhibit an indolent clinical course, whereas others have aggressive tumors that lead to death. Patients with ACC have not benefited much from the progress in oncological treatments developed for other tumors. The experience and knowledge accumulated in the past decade concerning the clinical outcomes and molecular pathogenesis of ACC highlight the impacts of heterogeneity at both molecular and clinical levels (3–6). Multiple studies in other cancer entities have shown that patients with higher tumor heterogeneity exhibit unfavorable clinical outcomes (7–11). Intratumor molecular heterogeneity is also regarded as a key factor that contributes to therapeutic failure and drug resistance (12). Therefore, inter- and intratumor heterogeneity are closely linked to typical tumor features responsible for poor patient outcomes (13). At present, the prognostic factors used in clinical practice for ACC are mainly reflected by the European Network for the Study of Adrenal Tumors (ENSAT) stage, as well as tumor proliferation indicator assessed either by mitotic count or Ki-67 (1, 14–16). Although combination of clinical and molecular markers has been proposed, it has not yet found general use (17, 18). Importantly, metabolomic changes that underlie the heterogeneous clinical course have not been identified, nor have particular tissue subpopulations that drive disease progression been uncovered.

MALDI mass spectrometry imaging (MALDI-MSI) allows label-free semiquantitative detection of numerous molecules in biological samples without prior knowledge of their presence or available high-resolution spatial information concerning their distributions. This capability is important for assessments of complex adrenal diseases, such as adrenocortical tumors (19). Thus far, MALDI-MSI has been used for metabolic profiling of endocrine tissues, as well as the visualization of hormone and metabolite distributions in normal and diseased adrenal glands (20, 21). Based on its ability to improve the understanding of the functional anatomy of the human adrenal glands (22), MALDI-MSI has been used to identify novel biomarkers and pathways associated with malignancy in ACC (23). This technique has also been used to explore genotype/phenotype correlations in patients with aldosterone-producing adenoma (24), pheochromocytoma, and paraganglioma (25). An improved understanding of the molecular features underlying tumor heterogeneity requires the identification and comprehensive molecular characterization of tumor subpopulations adversely affecting patient outcome. MALDI-MSI is undergoing rapid optimization to facilitate its application in biological research and clinical practice (26–29). Balluff et al. (30) and Bien et al. (31) used MALDI-MSI in combination with clustering algorithms to discover de novo phenotypic tumoral heterogeneity, which facilitated the identification of tumor subpopulations associated with adverse clinical outcome of patients with gastric cancer and breast cancer. Here we applied a similar approach in ACC to assess tumor metabolic heterogeneity.

*K*-means clustering is a common unsupervised machine learning algorithm used for clustering and grouping data points into distinct clusters. It can be used to identify different subtypes of cancer cells within a tumor based on various features, such as gene expression (32). Simpson’s diversity index (33) is a commonly used index for assessing diversity and richness. Based on MALDI-MSI data, we assessed de novo metabolic heterogeneity of tumor tissues from patients with ACC and identified tumor subpopulations via *k*-means clustering and Simpson’s diversity index. Additionally, we applied this pipeline to perform comparative analysis between 25 normal adrenal cortex samples and 72 ACC tumors, which revealed the common and unique subpopulations between normal cortex and ACC tumors.

## Results

### Schematic overview.

**Figure 1:**
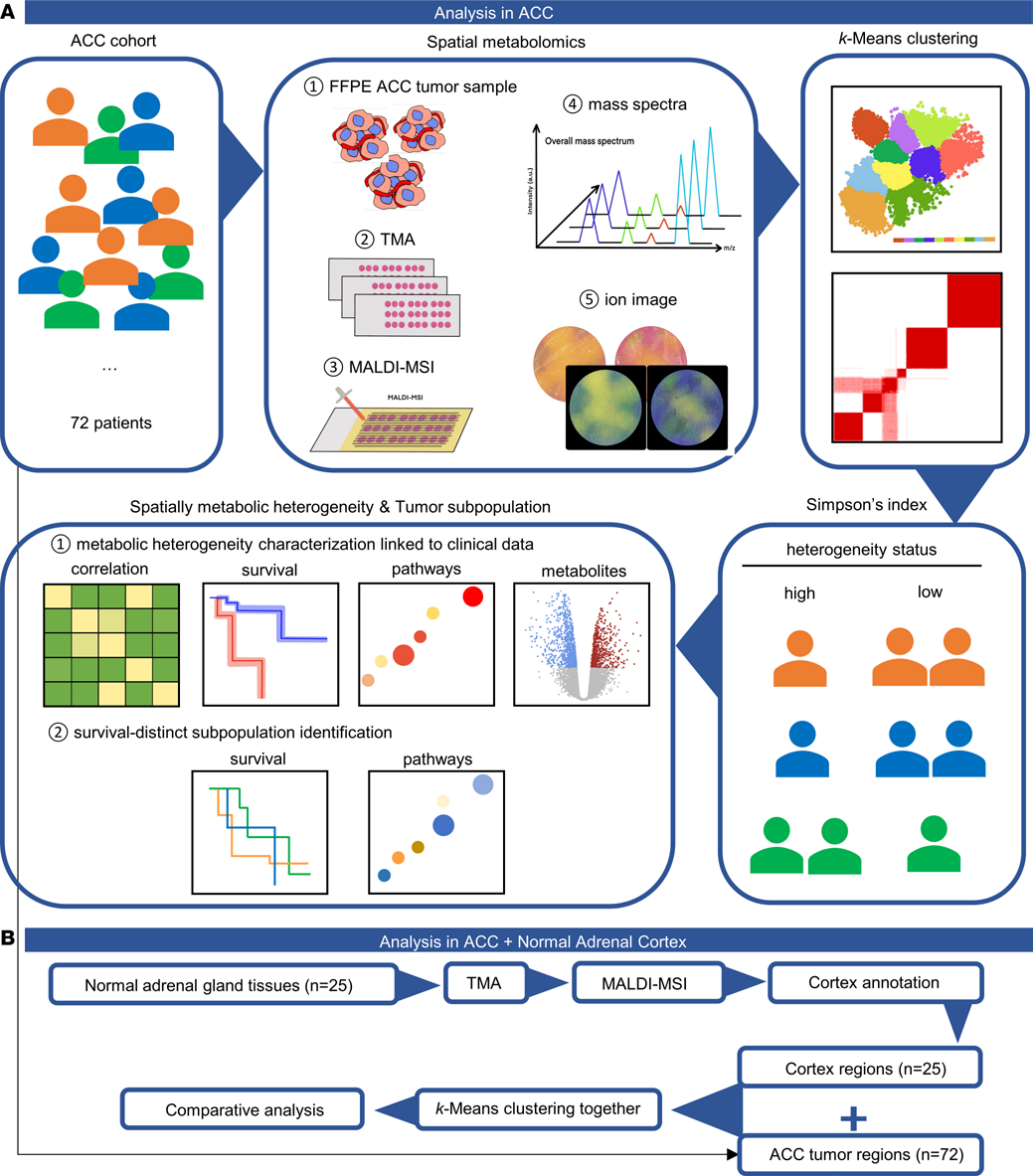
Schematic overview of the conceptual methodology for the de novo identification of metabolic heterogeneity and tumor subpopulations. (**A**) Workflow in 72 ACC tumor samples. The spatial metabolomics comprises TMA construction, matrix application, and MALDI-MSI measurement. The *k*-means clustering algorithm and Simpson’s diversity index calculation were applied to assess metabolic heterogeneity and identify tumor subpopulations, followed by bioinformatics analysis linking with data of clinical endpoints. (**B**) Workflow of comparison between 72 ACC tumors and 25 normal adrenal cortex samples. MALDI-MSI measurement was performed as in **A** after TMA construction of 25 independent normal adrenal glands. Adrenal cortex was annotated as ROIs for comparative analysis with ACC tumors. ACC, adrenocortical carcinoma; TMA, tissue microarray; MALDI-MSI, MALDI mass spectrometry imaging; ROIs, regions of interest.

A schematic overview of the conceptual methodology in this study is shown in Figure 1, comprising approaches used to assess metabolic heterogeneity and tumor subpopulations in 72 patients with ACC based on spatial metabolomics, *k*-means clustering, Simpson’s diversity index, and bioinformatics analysis linking to clinical data (Figure 1A), as well as the comparative analysis between 72 ACC patient samples and 25 additional normal adrenal cortex samples (Figure 1B). For the latter analysis, the medulla regions of all adrenal gland samples were excluded, and only cortex regions were included in further analysis. Detailed clinical characteristics are given in Table 1.

Within the mass range of *m/z* 50–1,000, approximately 2,500 individual mass spectrometry *m/z* species were resolved in tumor ROIs from 72 patients. Annotation of these 2,500 *m/z* species yielded 1,034 metabolites according to the Human Metabolome Database (https://www.hmdb.ca/); 362 metabolites had KEGG_id information.

### High intratumor metabolic heterogeneity is evident in tissues from patients with ACC.

**Figure 2:**
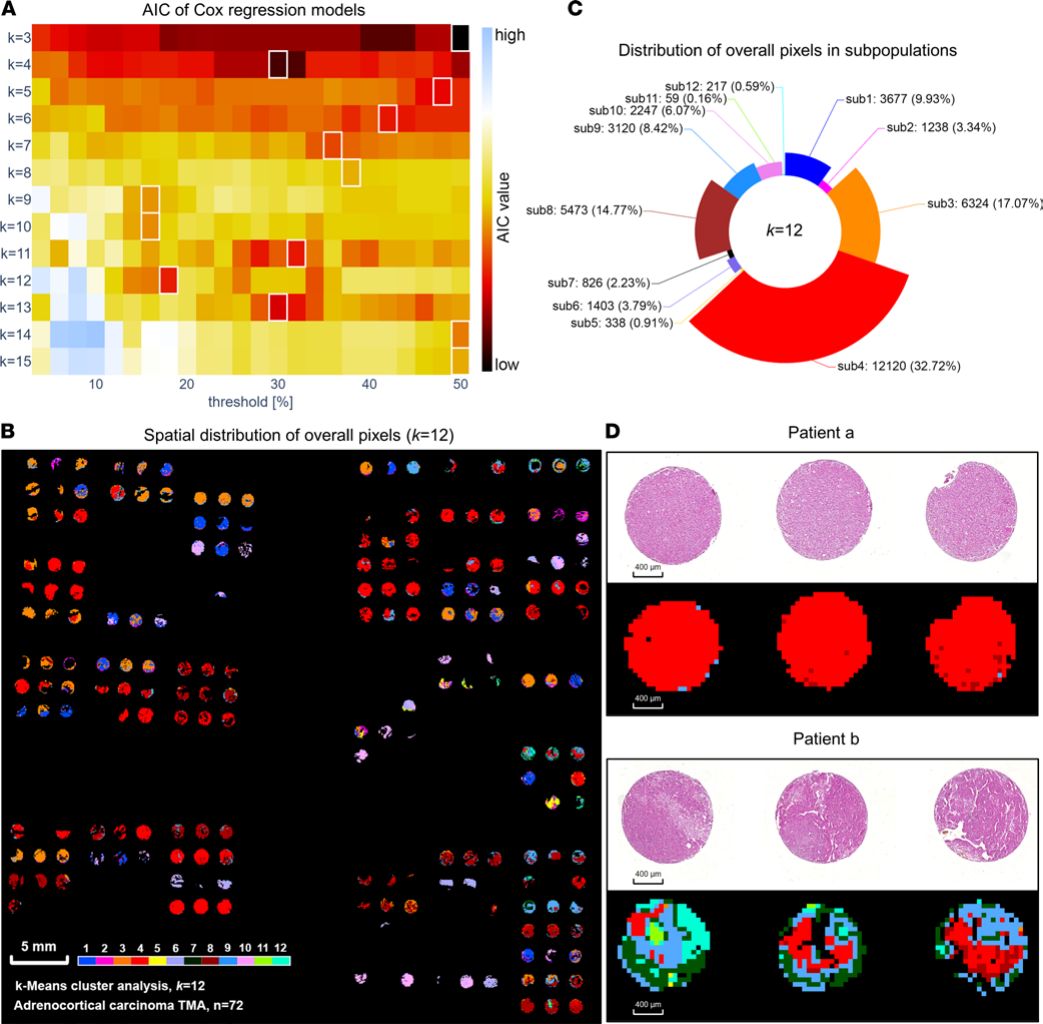
Spatial distribution of 12 tumor subpopulations identified in patients with ACC. (**A**) Heatmap of AIC threshold of each *k* value. Darker red indicates models with better fit; darker blue indicates models with poor fit, according to minimum AIC. White squares indicate lowest AIC value and best threshold at each *k*. (**B**) Distribution map of tumor subpopulations with *k* value of 12 in ACC tumors. Each color represents 1 tumor subpopulation. (**C**) Distributions of overall pixels in distinct tumor subpopulations. The central angle represents the percentage of pixels, the radius represents the number of pixels, and the number in parentheses indicates the proportion of pixels. (**D**) Examples of 2 patients show the distinct heterogeneity. More colors within the tissue cores represent more involved tumor subpopulations and therefore higher heterogeneity. ACC, adrenocortical carcinoma; AIC, Akaike’s information criterion; TMA, tissue microarray.

We first performed *k*-means clustering for *k* values of 2–15 among the 72 patients. Based on the clustering results, Akaike’s information criterion (AIC) was used to evaluate the fit of each *k* model (i.e., the patient distribution at each *k* value) by assigning distinct subpopulation presence thresholds that ranged from 4% to 50% (30); thresholds of 17%–50% produced optimal regression models (Figure 2A). The model of *k* = 2 was excluded because it exhibited very low variance; i.e., it completely determined whether a patient died or not. Three criteria were considered during model optimization. First, an increasing value of *k* was associated with greater similarity among patients in a single subpopulation. Second, a model with a lower AIC value more closely fit the data. Third, *P* values < 0.05 were indicative of statistically significant differences in survival. Although the models of *k* = 3 and *k* = 4 exhibited low AIC values, they did not meet the other criteria. Considering the above factors, *k* = 12 with a threshold of 19% was selected as the optimal and most informative model for further analyses. This optimal model exhibited higher stability and revealed significant differences in overall survival among tumor subpopulations.

Using a *k* value of 12, ROIs with molecular features were segmented into 12 tumor subpopulations (sub 1 to sub 12) as shown in Figure 2B. The spatial distribution of each subpopulation exhibited high metabolic heterogeneity within and between tumor tissue cores. The distribution of overall pixels revealed the greatest abundance in subpopulation 4 (32.72%) but the least abundance in subpopulations 11, 12, and 5 (sub 11 — 0.16%, sub 12 — 0.59%, and sub 5 — 0.91%) (Figure 2C). As examples of this segmentation, patients a and b contained 3 and 8 tumor subpopulations, respectively (Figure 2D).

### Simpson’s diversity index indicated variability in metabolic heterogeneity among patients with ACC.

**Figure 3:**
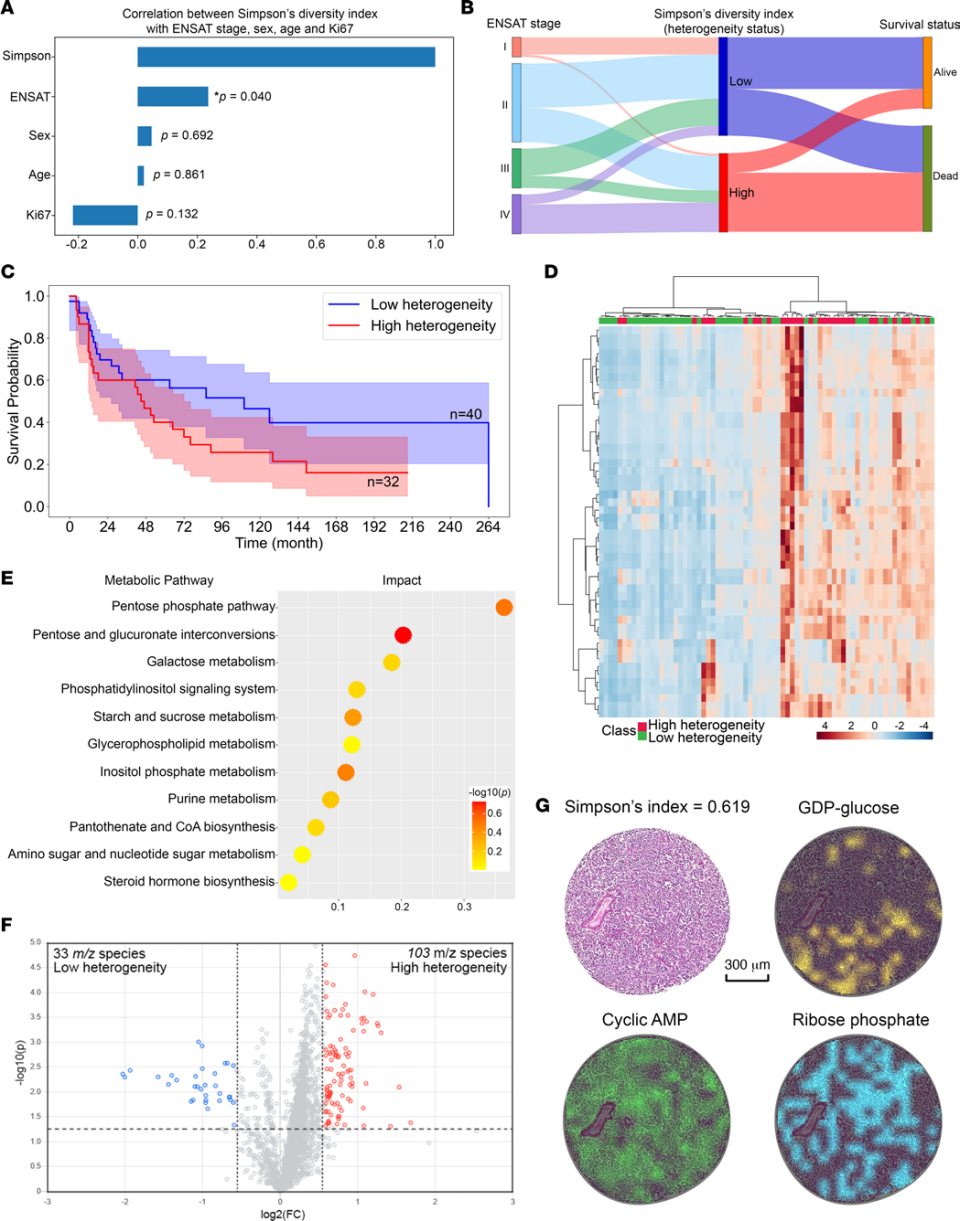
Metabolic heterogeneity in ACC based on Simpson’s diversity index. (**A**) Bar chart shows ENSAT tumor stage was positively correlated with metabolic heterogeneity determined by Simpson’s diversity index (i.e., higher Simpson’s diversity index indicates higher metabolic heterogeneity) (patients *n* = 72; *P* values were calculated by Pearson’s correlation for continuous values and Spearman’s correlation for ranked variables). (**B**) Distribution and flow of patients according to metabolic heterogeneity status and ENSAT stages, along with their survival status. The width is proportional to the number of patients. (**C**) Kaplan-Meier analysis–based patient stratification between low– and high–metabolic heterogeneity groups. Patients with low metabolic heterogeneity exhibited better survival. (**D**) Discriminative *m/z* species–focused heatmap clustering analysis reveals a distinct separation of patients with high (red) and low (green) metabolic heterogeneity. Columns represent patients (*n* = 72); rows represent *m/z* species (top 50). Color gradient represents the intensity from maximum (red) to minimum (blue). (**E**) Enriched pathways in patients with high metabolic heterogeneity. Each pathway is shown as a circle according to metabolic classifications and enrichment scores (vertical axis), as well as topology analyses (i.e., pathway impact; horizontal axis). Circle color represents the statistical significance of overall metabolic changes within each pathway. (**F**) Volcano plot of the distribution of differentially abundant *m/z* species, demonstrating 33 *m/z* species of increased (blue) abundance in low-heterogeneity patients versus 103 *m/z* species of increased (red) abundance in high-heterogeneity patients (fold-change cutoff = 1.5). The vertical dotted line indicates fold-change = 1.5, and horizontal dotted line indicates *P* = 0.05. (**G**) Heterogenous spatial distribution of 3 representative metabolites in a patient with high metabolic heterogeneity (the vessel region was excluded from analysis). ACC, adrenocortical carcinoma; ENSAT, European Network for the Study of Adrenal Tumors; GDP, guanosine diphosphate; AMP, adenosine monophosphate; FC, fold-change.

For further analysis of tumor heterogeneity among 72 patients, we considered the subpopulation number and size, then used Simpson’s diversity index to quantify metabolic heterogeneity in each patient, which ranged 0–0.798 (Supplemental Figure 1A; supplemental material available online with this article; https://doi.org/10.1172/jci.insight.167007DS1). By correlating to clinical data, we found a significant positive correlation between ENSAT tumor stage and metabolic heterogeneity represented by Simpson’s diversity index (Figure 3A) (*rho* = 0.242, *P* = 0.040). ENSAT stage IV exhibited significantly higher heterogeneity compared with ENSAT stage I (*P* = 0.026) (Supplemental Figure 1A). As illustrated in Figure 3B, patients at advanced ENSAT stage showed higher metabolic heterogeneity associated with unfavorable outcomes. Remarkably, a Sankey diagram revealed a nonlinear correlation of metabolic heterogeneity with ENSAT stages, particularly a trend for lower heterogeneity in ENSAT stage III compared with stage II. Following an optimized cutoff (0.49) for Simpson’s diversity index, Kaplan-Meier survival estimates indicated that patients with lower metabolic heterogeneity (*n* = 40) tended to survive longer, compared with patients who had higher metabolic heterogeneity (*n* = 32) (Figure 3C).

Further heatmap-based clustering analysis according to the discriminative *m/z* species revealed the separation of tumors with high and low metabolic heterogeneity (Figure 3D). The metabolites that contributed to heterogeneity stratification were used for pathway enrichment analysis (Figure 3E). The pentose phosphate pathway, pentose and glucuronate interconversions pathway, and galactose metabolism pathway were the most significantly altered pathways in tumors with high metabolic heterogeneity. Based on a fold-change threshold of 1.5, we identified 33 and 103 discriminative *m/z* species with increased abundance in patients with low and high metabolic heterogeneity, respectively (Figure 3F). Figure 3G shows the heterogeneous spatial distributions of 3 endogenous metabolites in highly heterogeneous ACC tumor tissue, among which ribose phosphate is a key metabolite in the pentose phosphate pathway.

To obtain insight into the relationship of tumor heterogeneity with tumor steroid hormone metabolites, we correlated metabolic heterogeneity to the presence of measurable tumor steroid hormone in patients with ACC, such as estrone 3-sulfate (E1S), estradiol-17β 3-sulfate (E2S), and estradiol-17β 3,17-disulfate (E2S2), and found a significant negative correlation between E1S with ACC tumor heterogeneity, similar to E2S (Supplemental Figure 1B).

### Certain tumor subpopulations are independent prognostic factors.

**Figure 4:**
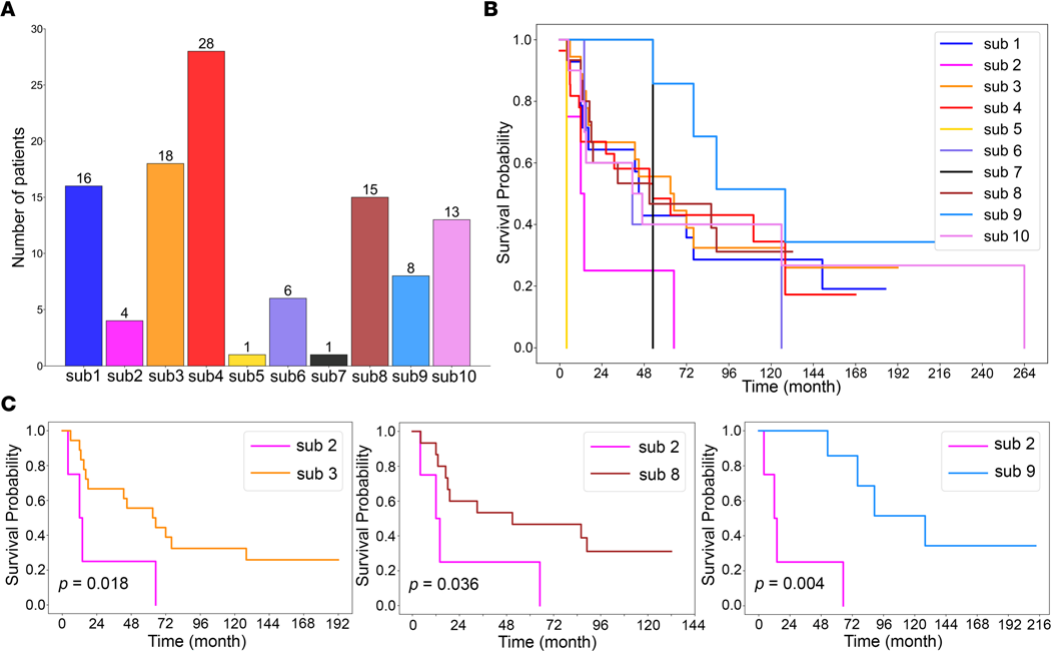
Tumor subpopulations impact survival of patients with ACC. (**A**) The number of patients assigned to each subpopulation according to minimum AIC threshold (2 subpopulations below the threshold were excluded). (**B**) Kaplan-Meier analysis of the survival according to subpopulation. (**C**) Sub 2 is a subpopulation associated with worse survival compared with sub 3, sub 8, and sub 9 (patients *n* = 4 in sub 2, *n* = 18 in sub 3, *n* = 15 in sub 8, *n* = 8 in sub 9; *P* values were calculated by Wald’s test). ACC, adrenocortical carcinoma; AIC, Akaike’s information criterion.

To explore the clinical impact of each tumor subpopulation, we linked the results of molecular segmentation to the patients’ clinical data. Using the AIC-based threshold of 19%, patients with a pixel percentage above the threshold were assigned to 10 tumor subpopulations; 2 subpopulations below the threshold were excluded (Figure 4A). The overall survival differences of patients in 10 tumor subpopulations are illustrated in Figure 4B. To determine which subpopulations are associated with specific prognoses, Kaplan-Meier analysis was performed in subpopulation pairs, which revealed statistically significant differences in overall survival between sub 2 versus sub 3 (*P* = 0.018), sub 2 versus sub 8 (*P* = 0.036), and sub 2 versus sub 9 (*P* = 0.004). Remarkably, sub 2 was associated with worse survival compared with sub 3, sub 8, and sub 9 (Figure 4C). To investigate the potential factors associated with prognosis, we performed Cox’s regression model with multivariate adjustment for tumor subpopulations, ENSAT stages, age, and sex that revealed the independent detrimental effect of sub 2, sub 4, sub 6, and sub 8 on overall survival (Table 2, sub 2: HR 7.610, 95% CI 1.867–31.012, *P* 0.005; sub 4: HR 4.617, 95% CI 1.767–12.066, *P* 0.002; sub 6: HR 6.300, 95% CI 1.341–29.598, *P* 0.020; sub 8: HR 2.624, 95% CI 1.005–6.854, *P* 0.049).

### Distinct tumor subpopulations are characterized by different pathways.

**Figure 5:**
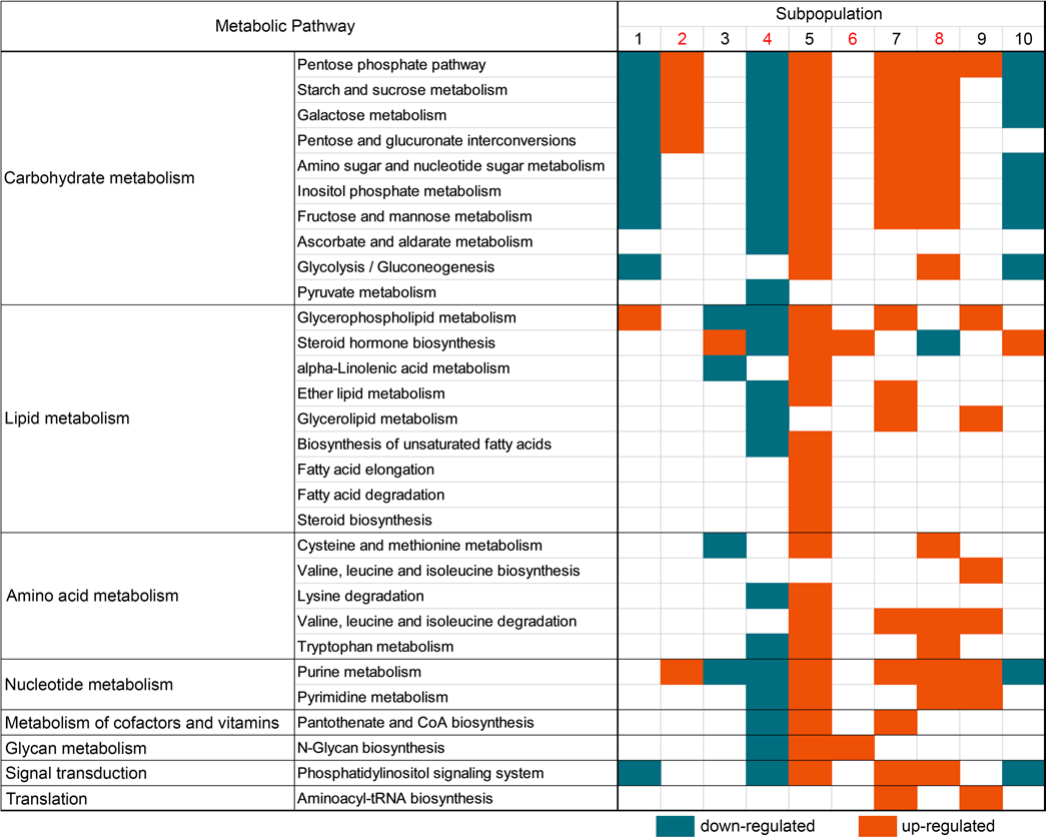
Comparison of metabolic pathways in ACC tumor subpopulations. Independent prognostic tumor subpopulations are indicated in red. Each dark cyan square indicates a downregulated pathway; each orange square indicates an upregulated pathway. ACC, adrenocortical carcinoma.

**Figure 6:**
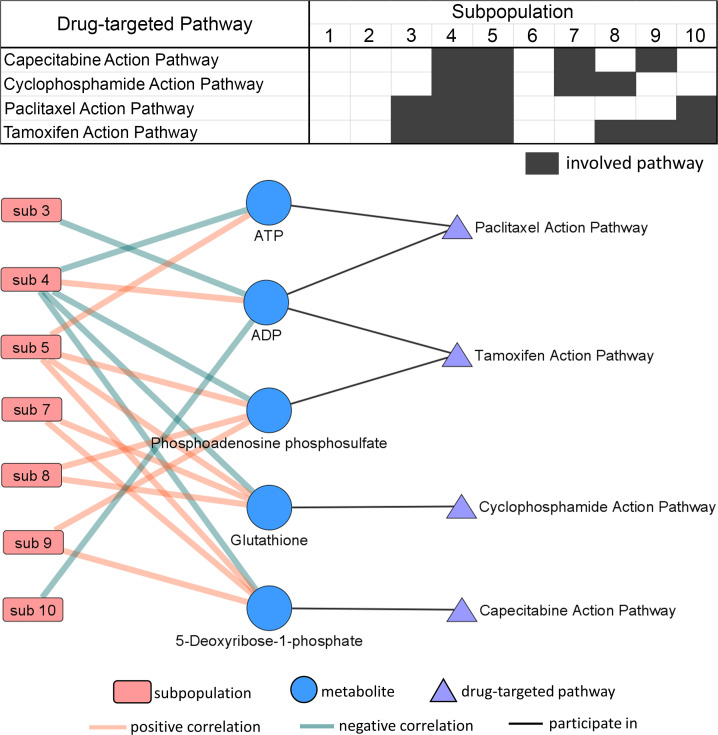
Association of anticancer drug–targeted pathways with different ACC tumor subpopulations. Each black square indicates that the drug pathway is present in a subpopulation. The network was generated with the correlated tumor subpopulations. The orange and cyan lines indicate positive or negative correlations, respectively, between metabolites and subpopulations. The black lines indicate that a metabolite plays a role in an anticancer drug pathway. ACC, adrenocortical carcinoma; ATP, adenosine triphosphate; ADP, adenosine diphosphate.

We selected differentially abundant metabolites in each subpopulation for pathway enrichment analysis. Figure 5 summarizes the discriminative metabolic pathways characteristic of corresponding subpopulations of patients with ACC. Classes of metabolites with variability were carbohydrate metabolism, lipid metabolism, and amino acid metabolism. The intratumor metabolic heterogeneity between distinct subpopulations was evident at the pathway analysis level. It is noteworthy that in the tumor subpopulations with independent prognostic value (sub 2, sub 4, sub 6, and sub 8), pentose phosphate pathway, starch and sucrose metabolism, galactose metabolism, pentose and glucuronate interconversions, and purine metabolism were enriched in sub 2 and sub 8, while those metabolic pathways were downregulated in sub 4 and not identified in sub 6. Interestingly, in sub 4, all identified pathways were downregulated, in contrast to the other 3 independent prognostic subpopulations. As demonstrated in Figure 6, 5 metabolites that play roles in 4 anticancer drug action pathways were discovered to closely correlate with tumor subpopulations, which indicated associations between tumor subpopulations with the drug action pathways of capecitabine, cyclophosphamide, paclitaxel, and tamoxifen. For example, the 4 drug pathways were all found to correlate with sub 4 and sub 5, while in sub 1, sub 2, and sub 6, there were no associated drug action pathways, which might indicate a worse response to chemotherapy in these 3 ACC tumor subpopulations.

### ACC tumor subpopulations share metabolomic features with normal adrenal cortex.

**Figure 7:**
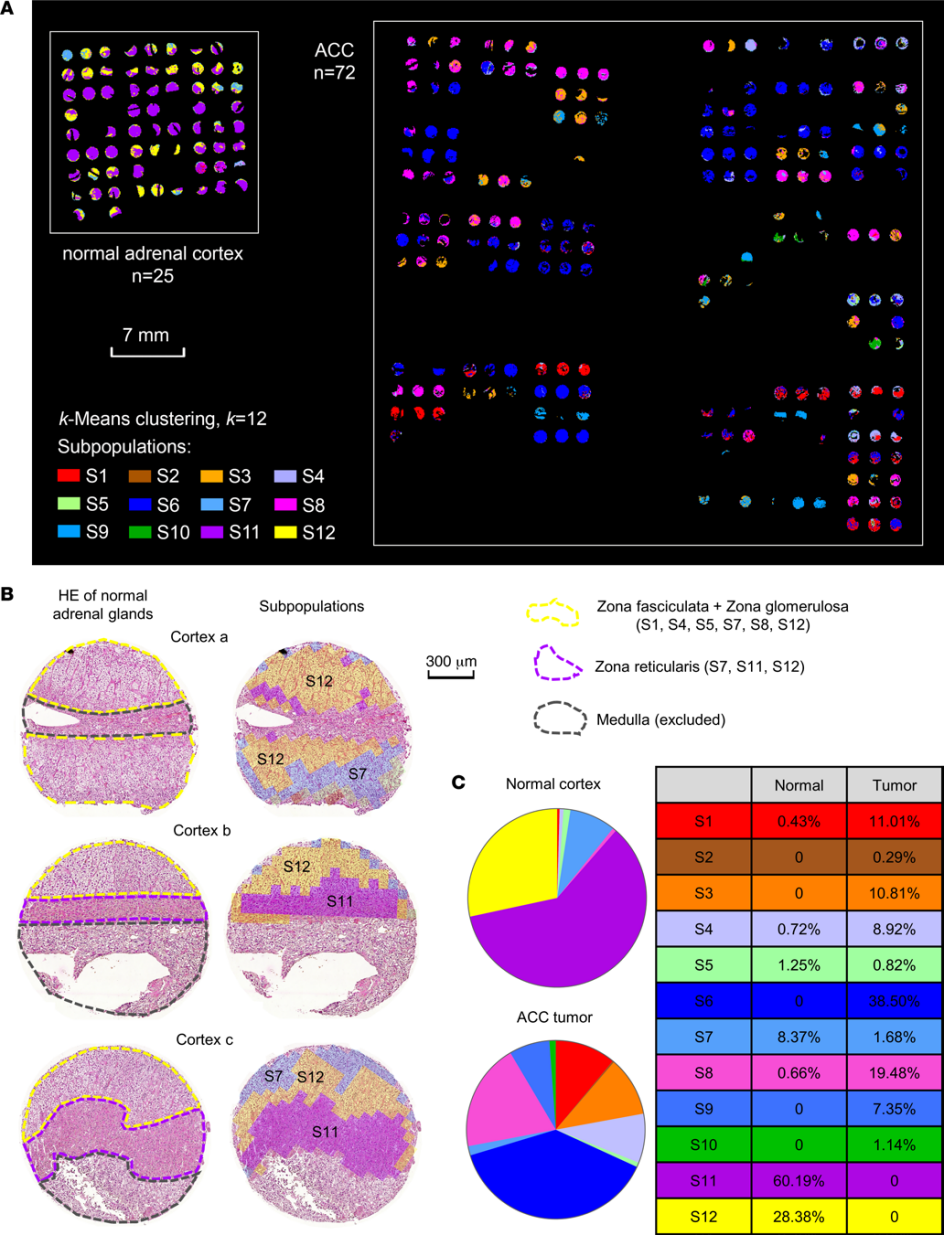
Subpopulations in combined de novo discovery of normal adrenal cortex and ACC tumors. (**A**) Visualization of 12 subpopulations (S1–S12) within 25 normal adrenal cortex and 72 ACC tumor ROIs via *k*-means clustering. (**B**) Examples of subpopulation distributions in 3 normal adrenal cortex tissue cores reflecting the functional anatomy in the cortex. (**C**) Pie charts show the presence of respective subpopulations in adrenal cortex and ACC tumors. ACC, adrenocortical carcinoma; ROIs, regions of interest; HE, hematoxylin and eosin.

**Figure 8:**
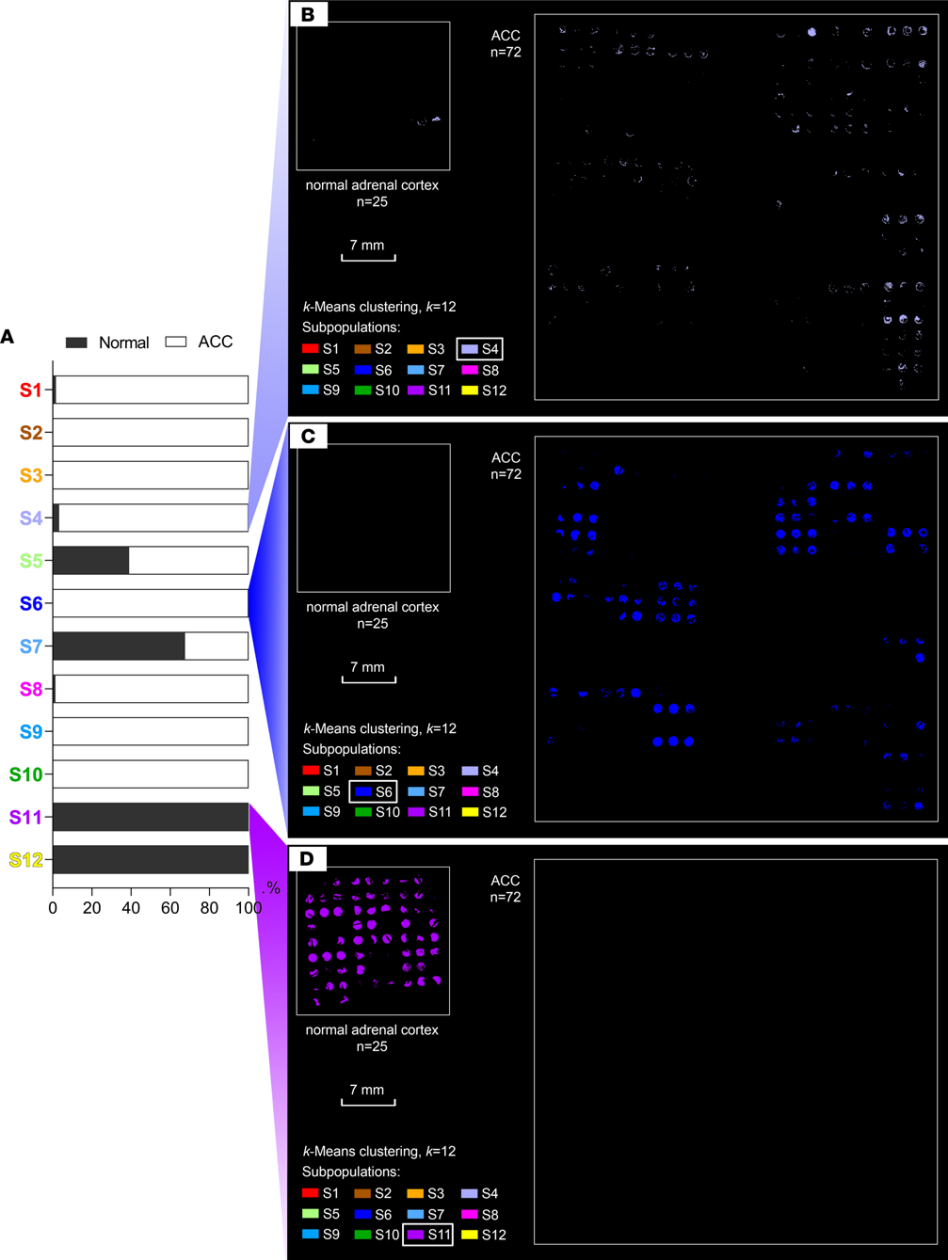
Visualization examples of 3 subpopulations. (**A**) Presence ratios of adrenal cortex (black bar) and ACC tumor (white bar) in each subpopulation. S4 (light purple) presented in both normal cortex and ACC tumor (**B**). S6 (blue) only presented in ACC tumor (**C**). S11 (purple) only presented in normal cortex (**D**). ACC, adrenocortical carcinoma.

To investigate the relevance of molecular pattern-based subpopulations present in normal adrenal cortex in comparison with ACC, we performed pixel-wise *k*-means clustering in 25 normal adrenal cortex and 72 ACC tumor regions in 1 run with *k* = 12. By doing so, we obtained a spatial segmentation map displaying 12 metabolic subpopulations (S1–S12) within normal adrenal cortex and ACC (Figure 7A). By coregistering with H&E-stained images, we found that most subpopulations showed high similarity with zona fasciculata and zona glomerulosa, such as S7 and S12, while several subpopulations showed high similarity with zona reticularis, particularly S11 (Figure 7B). By comparing the distributions of 12 subpopulations in normal adrenal cortex and ACC tumors, we were able to identify not only unique subpopulations in normal cortex or ACC tumors but also shared subpopulations by both normal and tumor (Figure 7C and Figure 8A). For example, S4 presented in both normal cortex and ACC tumors (Figure 8B), S6 presented as an ACC-specific subpopulation (Figure 8C), and S11 presented as a normal cortex–specific subpopulation (Figure 8D). However, the majority of ACC metabolic subpopulations were absent in normal adrenal cortex. These results verify the metabolic heterogeneity also existed in normal adrenal cortex. However, fewer subpopulations were detected in normal adrenal cortex, indicating a lower intensity in metabolic heterogeneity compared with ACC tumors.

## Discussion

In this study, we explored metabolic heterogeneity by spatial metabolomics in tissues from 72 patients with ACC and 25 normal adrenal glands for comparison. Thus far, various tumors have been reported to exhibit highly heterogeneous metabolic profiles that contribute to the connective metabolic networks within such tumors, as well as networks between the tumors and their surrounding environments. Tumor metabolic heterogeneity may influence tumor progression and patient outcomes, and an improved understanding of this metabolic heterogeneity may yield alternative clinical strategies (34). Our research not only revealed a variability in heterogeneity within ACC but also was able to identify distinct tumor subpopulations of which the presence of certain ones was independent of prognostic relevance. Some tumor subpopulations were discovered to associate with different anticancer drug action pathways. In addition, we found several ACC tumor subpopulations to be shared with zona reticularis of normal adrenal cortex, suggesting its histogenetic origin there.

Despite the generally unfavorable prognosis of ACC, there is variation among patients in terms of progression, recurrence, and overall survival. Basic research and clinical studies of ACC have enhanced the understanding of this disease and enabled assessments of genetic heterogeneity during tumor progression. Some pan-genomic studies have identified features closely associated with prognosis (35, 36) and proposed targeted molecular markers for the prognostic assessment of ACC (37–39). These molecular markers were identified at tumor DNA or RNA level. In a small series of 14 ACCs that underwent exome sequencing, intratumor heterogeneity was reported in 43%–63% of somatic mutations among different metastatic sites from the same patient (40). Jouinot et al. (18) assessed the robustness of targeted molecular markers measurable at the DNA level in 26 patients with ACC. The results indicated that intratumor heterogeneity affects DNA-related molecular markers. At variance, prognostic DNA methylation patterns as well as chromosome alteration profiles appear rather stable and might be more robust for ACC prognostic assessment.

MSI renders the phenotypic consequences of genetic alterations accessible and allows for spatial information that may enable an improved understanding of the complex factors that affect cancer reprogramming. These may be used for prognostic assessment and improved treatment. Here, the analysis of 72 patients with ACC discovered extensive metabolic heterogeneity within and between individual tumor samples, which suggests its biological relevance by demonstrating association with survival. Tumor-related excess of steroid hormones such as cortisol and sex steroids is the leading clinical finding in 50%–60% of cases of ACC (41, 42). Previously, Sun et al. (23) investigated the role of tumoral steroid hormone metabolites in ACC and reported that high abundance of E1S was significantly associated with more favorable prognosis similar to E2S whereas the presence of E2S2 was associated with particularly poor overall survival. In order to test for the relationship between metabolic heterogeneity and tumor steroid excess, we correlated heterogeneity with steroid abundance and found them to be negatively associated, in line with our finding that patients with high metabolic heterogeneity exhibited worse prognosis.

Tumor metabolic heterogeneity results from internal and external factors (43), both of which are heterogeneous and contribute to metabolic heterogeneity through the activation of specific signaling pathways that induce distinct metabolic responses (43–45). Although genetic variability has been a long-term focus of tumor research (46), it has been challenging to address the lack of predictability concerning spatial and temporal heterogeneity (47). Analyses of metabolic heterogeneity may overcome the technical limitations of other tools, providing clinical insights regarding tumor metabolism (48). Within samples from 72 patients with ACC, we defined 10 distinct tumor subpopulations at the metabolomics level. Four out of 10 tumor subpopulations were found to be independent prognostic factors — sub 2, sub 4, sub 6, and sub 8. Sub 2 was associated with a particularly unfavorable prognosis. Metabolic reprogramming is a common hallmark of human cancers, with important implications for tumor progression and patient survival (49). Numerous challenges persist in targeting these metabolic alterations because of metabolic tumor heterogeneity (47). Differential pathway utilization was observed even between the different subpopulations, with enhanced activity of the pentose phosphate pathway observed in sub 2, sub 8, and patients with high metabolic heterogeneity. This is in line with the idea that activation of the pentose phosphate pathway directly contributes to cell proliferation, survival, and senescence. In addition, the pentose phosphate metabolite phosphoribosyl pyrophosphate is important for the formation of purine nucleotide. In the present study, purine metabolism was also found to be upregulated in sub 2 and sub 8. Previous studies (50) have demonstrated high concentrations of purine metabolites in tumor cells and concluded that purine metabolism may be an attractive cancer treatment strategy. Our findings provide evidence to support this conclusion and a clue to improved prognosis of heterogeneous ACC subpopulations.

Drug repurposing has been proposed as an effective shortcut to drug discovery. For ACC treatment, mitotane is the only FDA-approved drug (51–54) and currently used both in postoperative adjuvant and palliative (advanced) care settings. The polychemotherapy regimen etoposide, doxorubicin, and cisplatin plus oral mitotane represents the current standard of chemotherapy for advanced ACC, being the only treatment strategy supported by a randomized controlled trial. We identified actionable pathways of 4 anticancer drugs including capecitabine, cyclophosphamide, paclitaxel, and tamoxifen in several ACC tumor subpopulations. Interestingly, sub 2 was not associated with a specific drug pathway, which might reflect poor treatment responsiveness of this specific subpopulation. Although capecitabine has been used for salvage therapy of ACC in combination with gemcitabine (55, 56), cyclophosphamide, paclitaxel, and tamoxifen are not commonly used for ACC treatment. In our study, 3 ACC subpopulations, including sub 5, sub 7, and sub 9, were positively correlated to the capecitabine action pathway and might be considered to respond to this treatment, considering that sub 5 and sub 7 contained only 1 patient each. Paclitaxel has been studied in combination with sorafenib (57) but was largely inactive. In the current study, the action pathway of paclitaxel displayed an association with independent prognostic factor sub 4. Paclitaxel could work especially for this tumor subpopulation. Taken together, metabolic features of ACC could be associated with preferential activity of chemotherapy regimens, opening alternative therapeutic opportunities.

To date, only a few papers mainly focused on comparison of normal adrenal glands and ACC in humans. Predictably, when compared with the normal adrenal cortical samples, ACC samples exhibited the hallmark features of neoplastic tissue. Our results discovered a major metabolic difference of normal adrenal cortex from ACC within the overall metabolic profile. They are in agreement with the findings of Imperiale et al. (58), who reported a clear separation between normal adult human adrenal gland and adrenal cortical pathologies, such as ACC, based on specific metabolic fingerprints. In addition, the observations within normal adrenal cortex–specific subpopulations seemed to indicate their correspondence to normal anatomical structures. Unfortunately, the number of the samples analyzed does not allow us to draw any definitive conclusion.

A major study limitation is the number of patients included. Due to the limited sample size, we did not adjust *P* values for multiple testing. Nevertheless, our study cohort should be considered large given the rarity of ACC. Our workflow included a multifactorial approach to determine the optimal number of tumor subpopulations and the threshold for classifying patients into high- or low-heterogeneity groups. The definitions of the threshold and the optimal number of clusters were based on *P* values. Alternatively, the selection procedure could use other methods, such as cluster analysis. Our approach, following Balluff et al. (30), has been improved by applying an extended Cox regression model that includes the count process formulation of Andersen and Gill (59) when comparing subpopulations to account for situations where patients belong to more than 1 subpopulation. The use of TMAs has both benefits and limitations. TMAs allow for the measurement and analysis of multiple tissue samples under the same condition, which can lead to more comparable and robust results. Nonetheless, while all tissue cores were identified as representative regions by experienced pathologists, they do not represent entire tumors. Despite this, we followed standard and well-established protocols for working with TMAs. Our study could be expected to be the basis for analysis in a larger patient series and extended clinical follow-up.

The present study investigated the heterogeneity of metabolic profiles in ACC and pinpointed tumor subpopulations associated with worse survival and certain anticancer drug action pathways. The findings illustrated the potential for combining MALDI Fourier transform ion cyclotron resonance MSI (FT-ICR-MSI) and advanced statistical clustering approaches to explore metabolic heterogeneity in ACC. Detailed insights into tumor heterogeneity and information concerning changes in tumor subpopulations might help develop clinical strategies for ACC management.

We herewith present a study that uses MALDI-MSI–based spatial metabolomics to investigate the metabolic heterogeneity and tumor subpopulations in ACC. The results revealed variability in ACC heterogeneity and several survival-distinct ACC subpopulations, which also differed at the level of pathway enrichment. Our findings complement genetic and gene expression data and may aid in the identification of targetable cancer pathways.

## Methods

### Spatial metabolomics experiments.

Briefly, the FFPE tissue samples from 72 patients with ACC received from the ENSAT registry (https://registry.ensat.org) were transferred into 6 TMAs. None of the patients received systemic treatment prior to tissue sampling. Each patient presented with 3 cores of 1 mm diameter. All the cores were from the representative tumor areas identified by experienced pathologists. Similarly, the FFPE tissue samples from 25 independent human normal adrenal glands were obtained and transferred into 1 TMA. All TMA samples were cut into 3 μm–thick sections using a microtome (HM 355S, Microm, Thermo Fisher Scientific) and mounted onto indium tin oxide–coated glass slides. A SunCollect automatic sprayer (SunChrom) was used for the application of a 9-aminoacridine hydrochloride monohydrate (MilliporeSigma) matrix. MALDI-MSI analyses were performed on a solariX 7T FT-ICR mass spectrometer (Bruker Daltonics) operating in negative ion mode, followed by tissue section staining with H&E. More details about the experiments are in our previous report (23). The MALDI FT-ICR-MSI data were acquired over a mass range of *m/z* 50–1,000 with a 60 μm lateral resolution, then subjected to spectral processing using SCiLS Lab 2021c (Bruker Daltonics) after a coregistration of optical images. For further analysis, only the tumor regions of ACC samples and the cortex regions of adrenal glands were annotated as ROIs.

### Unsupervised k-means clustering.

The *k*-means clustering method was initially used to identify sets that were spectrally similar but not necessarily spatially adjacent (clusters). Because of uncertainty regarding the extent of heterogeneity, we performed the primary analysis for *k* values of 2–15 within ROIs containing resolved mass spectrometry peaks. Segmentation clustering was conducted using the segmentation tool in SCiLS Lab (parameters: Normalization_Root Mean Square, Method_*k*-Means, #Classes_2-15) to reveal the spatial distribution of tumor subpopulations by displaying distinct metabolic regions (i.e., clusters) in different colors.

### Simpson’s diversity index.

Simpson’s diversity index (33, 60), which denotes the probability that 2 randomly chosen pixels are from different types and measures diversity, is defined as follows:


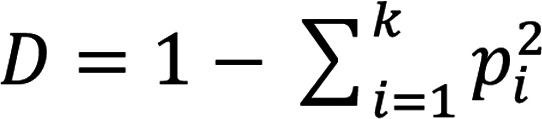
, where *p_i_* is the share of pixels in cluster *i*, and *k* is the number of clusters.

The index, computed for each patient, can have values between 0 and 1, with 0 indicating that all the pixels from 1 patient are in 1 cluster, and the higher the values of the index, the higher the diversity of the pixels in the different clusters for a patient. The calculation was performed in R (version 4.0.4).

### Optimization of cluster presence threshold.

The statistical analysis required linking patient survival data to the presence of specific clusters (tumor subpopulations). In this process, a patient was assigned to a cluster if the cluster was sufficiently present in that patient (i.e., if the cluster contained a fraction of pixels above a particular threshold); the cluster presence threshold was established based on this “sufficient presence.” A single patient could be assigned to more than one cluster if that patient’s tumor exhibited significant heterogeneity. The effect of threshold variation on survival was investigated using Cox’s proportional hazards regression models. Specifically, an iterative loop was created with thresholds ranging from 2% to 50%. At each threshold, a binary variable was created by applying the threshold to the cluster ratio. A Cox proportional hazards regression model was then built using the thresholded data. The quality of each regression model was evaluated using the AIC, which provides an assessment of each model’s fitness, where the model with the lowest AIC value was presumed to most closely fit the data. The analyses were repeated over *k* values of 2–15. Additional details regarding the application of the model were previously published (30). By comparing the presence percentages of 72 ACC patients to the determined AIC threshold, the patients with a sufficient percentage were assigned to 12 tumor subpopulations accordingly, and the patients with a percentage below the threshold were excluded from the corresponding tumor subpopulations.

### Survival estimates.

After calculation of the Simpson diversity index, we used a Kaplan-Meier survival curve to separate patients with low and high metabolic heterogeneity into 2 groups. An optimal threshold for low and high metabolic heterogeneity was chosen according to the minimal *P* value in the log-rank test.

Multivariate Cox’s proportional hazards model was performed to evaluate simultaneously the effect of AIC-thresholded tumor subpopulations on survival — including subpopulations containing more than one patient — ENSAT tumor stages, age, and sex. In Kaplan-Meier survival curve analysis, an extended Cox regression model was used to evaluate the statistical differences of the survival of subpopulations. The extended Cox regression model incorporates the count process formulation of Andersen and Gill to manage the possibility that some patients belonged to various subpopulations (59). Differences in survival were determined with the Wald test.

### Bioinformatics analysis.

We generated a heatmap-based clustering and volcano plot (fold-change ≥ 1.5, *P* < 0.05) using MetaboAnalyst database (https://www.metaboanalyst.ca/). The Kyoto Encyclopedia of Genes and Genomes (KEGG) database (http://www.genome.jp/kegg/) and MetaboAnalyst 5.0 were used to investigate metabolic pathways. The Small Molecule Pathway Database 2.0 (https://www.smpdb.ca/) was used to investigate drug-targeted pathways. To identify discriminative pathways characteristic of subpopulations, the Pearson correlation coefficient (*r*) was first calculated within the peak intensities and subpopulation percentages to identify peaks that were significantly correlated with each subpopulation. The peaks that were positively and negatively correlated with each subpopulation were then mapped onto the respective KEGG pathways to identify upregulated and downregulated pathways, followed by categorization into major metabolite classes.

### Statistics.

Distributions of Simpson’s diversity index between ENSAT stages were pairwise compared via unpaired 2-tailed Mann-Whitney *U* test. Relations between variables were assessed using Spearman’s correlation coefficient (*rho*) for ranked cofactors and Pearson’s correlation coefficient (*r*) for continuous values. All the *P* values are nonadjusted in this study because of the limited sample size. A *P* < 0.05 was considered statistically significant in all analyses.

### Study approval.

This study was conducted with approvals from the Ethics Committee of the University of Wuerzburg (approval numbers 86/03 and 88/11, Wuerzburg, Germany) and Klinikum der Universität München (approval numbers 379/10, Munich, Germany).

### Data availability.

The raw spectra data that support the findings of this study are available online (http://figshare.com/, retrieve code: 10.6084/m9.figshare.22700002; https://figshare.com/articles/dataset/MALDI-MSI_spectra_in_adrenocortical_carcinoma_tissues/22700002). Values for all data points found in graphs are in the Supporting Data Values file.

## Author contributions

AW, NS, and QW conceived the study; QW performed formal analysis, data curation, and manuscript writing; TK, NS, and AF performed MALDI-MSI experiments; SK, UD, IW, MF, and FB performed clinical data acquisition; QW performed statistical analyses; RM, RLG, and QW performed Simpson’s diversity index calculation; QW, MK, AW, NS, RM, RLG, MF, JW, and JS performed manuscript review and editing; AW and NS supervised the project; and AW, MK, and QW performed project administration and funding acquisition. All authors approved submission of the manuscript.

## Supplementary Material

Supplemental data

Supporting data values

## Figures and Tables

**Table 1:**
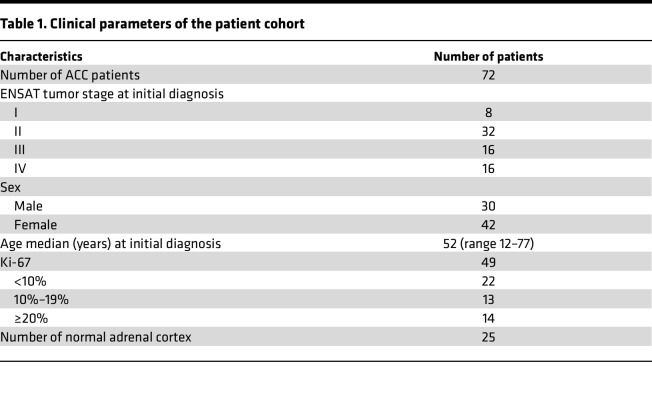
Clinical parameters of the patient cohort

**Table 2:**
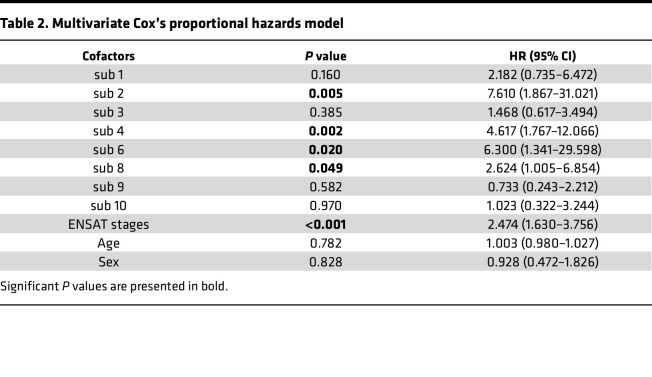
Multivariate Cox’s proportional hazards model

## Version 1 08/22/2023

Electronic publication
