# Supplementary material for: Metabolic heterogeneity in adrenocortical carcinoma impacts patient outcomes
Source: JCI Insight. 2023 Aug 22;8(16):e167007. doi: 10.1172/jci.insight.167007 (PMC10543722; doi:10.1172/jci.insight.167007)

Supplementary data for

Metabolic Heterogeneity in Adrenocortical Carcinoma Impacts Patient Outcomes

Qian Wang*, Na Sun*, Raphael Meixner, Ronan Le Gleut, Thomas Kunzke, Annette Feuchtinger, Jun Wang, Jian Shen, Stefan Kircher, Ulrich Dischinger, Isabel Weigand, Felix Beuschlein, Martin Fassnacht, Matthias Kroiss** and Axel Walch**

*These authors share the first authorship.

**These authors share the last authorship

****Correspondence to:**

Prof. Axel Walch

Research Unit Analytical Pathology, Helmholtz Zentrum München, Ingolstädter Landstraße 1, 85764 Neuherberg, Germany

E-mail: axelkarl.walch@helmholtz-munich.de

Prof. Matthias Kroiss

Department of Internal Medicine IV, LMU Hospital, Ludwig-Maximilians-Universität München (LMU), Ziemssenstrasse 5, 80336 Munich, Germany

E-mail: Matthias.Kroiss@med.uni-muenchen.de

Figure S1. (A) Distributions of Simpson's diversity index in patients at different ENSAT tumor stages identified that ENSAT stage IV displayed higher metabolic heterogeneity than ENSAT stage I (patients total n = 72; *p* = 0.026 by Mann-Whitney test). **(B)** Correlation between metabolic heterogeneity with tumoral steroid hormone metabolites (*p*-values were calculated by Pearson's correlation). (E1S - estrone 3-sulfate, E2S - estradiol-17ß 3-sulfate, E2S2 - estradiol-17ß 3,17-disulfate)

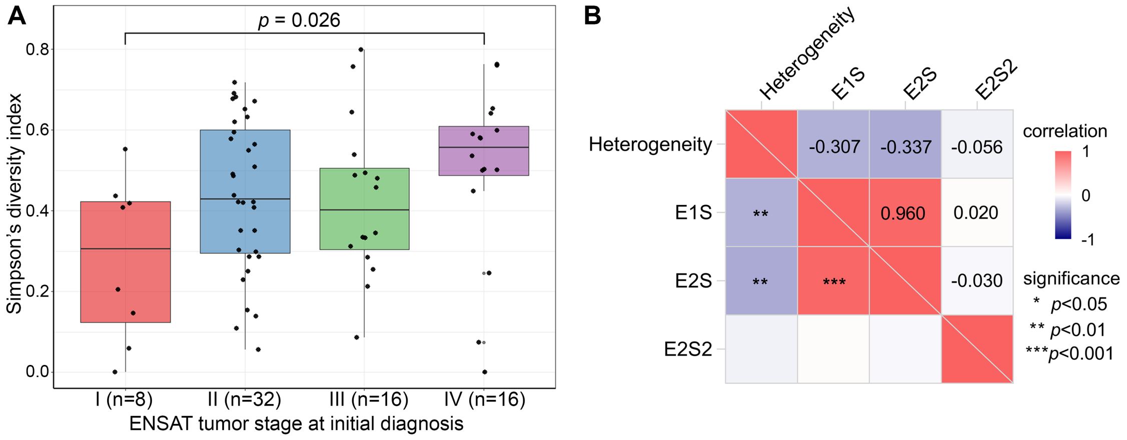

Supplement: Supplemental data [file jciinsight-8-167007-s012.pdf]
